# Unlocking the depths: the evolution of robotic-assisted bronchoscopy

**DOI:** 10.3389/fonc.2025.1546430

**Published:** 2025-03-10

**Authors:** Juliana Guarize, Luca Bertolaccini

**Affiliations:** ^1^ Division of Interventional Pulmonology, IEO, European Institute of Oncology IRCCS, Milan, Italy; ^2^ Department of Thoracic Surgery, IEO, European Institute of Oncology IRCCS, Milan, Italy

**Keywords:** robotic bronchoscopy, lung cancer, real-world applications, bronchoschopy, ion

## Introduction

Robotic-assisted bronchoscopy (RAB) has emerged as a transformative modality in diagnosing peripheral pulmonary lesions, overcoming the limitations of traditional diagnostic techniques. Among the pioneering platforms is the ION™ endoluminal system (ION) by Intuitive Surgical (Sunnyvale, CA, US), which has garnered significant attention for its innovation and clinical impact. This opinion article explores the ION platform’s cutting-edge advancements, real-world applications, and prospects, situating it at the forefront of interventional pulmonology.

## A revolution in diagnostic accuracy

The RAB system leverages shape-sensing robotic-assisted technology to provide unparalleled access and precision in navigating complex bronchial pathways. Previous studies in the Literature have demonstrated how this technology enhances navigation accuracy, particularly in reaching peripheral pulmonary lesions, thereby improving diagnostic yield and clinical outcomes (1, 2). This capability has addressed one of the longstanding challenges in bronchoscopy: reaching small peripheral lesions with high diagnostic yield. Unlike traditional bronchoscopes or previous electromagnetic navigational bronchoscopy (ENB) systems, which rely on pre-procedural computed tomography (CT) scans susceptible to CT-to-body divergence, RAB platforms offer real-time navigation with enhanced stability and control. CT-to-body divergence refers to the discrepancy between the lesion’s location in a pre-procedural CT scan and its actual position during bronchoscopy, often due to respiratory motion or anatomical shifts. This phenomenon can reduce the accuracy of navigation systems, particularly those relying solely on static imaging. RAB has improved access to pulmonary parenchymal nodular opacities, allowing for more accurate sampling. This distinction is critical, as not all thoracic nodules are pulmonary in origin. This advancement has improved the feasibility of accessing small peripheral lesions, though challenges remain across different technologies (3, 4).

A recent multicenter feasibility study in China demonstrated that the ION system can biopsy peripheral pulmonary lesions with a remarkable diagnostic yield of 87.8% (5). This aligns with findings from a US-based retrospective comparison, where RAB, using the ION platform, achieved an 86.1% diagnostic yield, outperforming ENB’s 49.5% yield (2). The system’s ability to access small lesions with high precision underscores its utility in early lung cancer detection, where timely and accurate diagnosis can significantly improve patient outcomes (2, 3).

## Enhanced safety profile

The RAB system’s advancements are not limited to diagnostic accuracy but extend to patient safety. Traditional techniques such as transthoracic needle aspiration pose risks like pneumothorax and hemorrhage (6, 7). Conversely, the ION platform integrates radial endobronchial ultrasound to confirm lesion localization and employs fine motor control to reduce iatrogenic complications (5). Studies in the Literature report a pneumothorax rate as low as 1.1%, with no cases requiring intervention, highlighting its safety even in high-risk scenarios (1).

Additionally, the platform’s compatibility with adjunctive imaging modalities, such as cone-beam CT, enhances tool-in-lesion confirmation and procedural success. Cone-beam CT-guided RAB significantly increased the diagnostic yield while maintaining safety standards, particularly by improving the accuracy of lesion targeting during navigation (8). In addition, cone-beam CT-guided ION significantly improves sampling accuracy without exceeding safety thresholds for radiation exposure (9). Such integration is pivotal in minimizing diagnostic errors and ensuring procedural efficacy (10).

## Comparative superiority

While the ION system shares the RAB landscape with competitors like the Monarch™ platform (Auris Health, Johnson & Johnson, US), it distinguishes itself through unique technological features (**Table 1**). The ION system’s shape-sensing catheter provides continuous positional feedback, enabling precise navigation even in anatomically challenging airways (3, 6). In contrast, Monarch™ is more prone to inaccuracies from dynamic airway changes (10). The superiority of the ION is evident in its higher diagnostic yields across lesion sizes and its ability to maintain stability during tool insertion and retraction. These capabilities have positioned it as the preferred choice for many interventional pulmonologists (10). Moreover, the platform’s real-time 3D imaging and integrated planning software streamline pre-procedural mapping, reducing the risk of CT-to-body divergence (2, 3).

**Table 1:** Comparative features of the robotic-assisted bronchoscopy platforms.

Feature	ION™ System	Monarch™ Platform
Navigation Technology	Shape-sensing robotic	Electromagnetic guidance
Diagnostic Yield	Up to 87.8%	Approximately 70%
Lesion Accessibility	Superior for peripheral lesions	Good for central lesions
Integration with Imaging	Real-time cone-beam CT compatibility	Fluoroscopy
Safety Profile	Low pneumothorax risk	Comparable

CT, computed tomography.

## Expanding applications

The success of the RAB in diagnosis has prompted exploration into therapeutic applications. A significant advantage of ION is the potential for a single surgical event, where diagnosis, operative localization, and resection are integrated into one procedure. This is further facilitated by fiducial marker placement, allowing for precise tumor targeting and enhanced surgical planning. Studies have highlighted the unique ability of the ION system to maintain positional accuracy and deliver therapeutic interventions in peripheral lung lesions (2, 10). Ongoing trials, and further investigating is being done on its utility in combining diagnostic precision with targeted ablation therapies, positioning it as a versatile tool for future therapeutic strategies (11). Such advancements could redefine lung cancer management by enabling minimally invasive treatments alongside diagnostic procedures, fulfilling the vision of a “one-stop shop” for pulmonary care (12, 13).

## The future of lung cancer treatment with robotic-assisted bronchoscopy

The arrival of the RAB heralds a new era in thoracic surgery with profound implications for the field. The ION system’s advanced navigation and precise localization capabilities enhance preoperative planning and intraoperative guidance (8, 9, 14). These features allow surgeons to pinpoint and access challenging nodules. Additionally, its potential integration with therapeutic modalities, including targeted drug delivery and molecular diagnostics, highlights its transformative impact on thoracic surgery practices. The platform paves the way for multidisciplinary lung cancer care by integrating advanced diagnostic capabilities with therapeutic potential (15). Interventional pulmonologists, advanced diagnostic bronchoscopists, and thoracic surgeons can now achieve earlier, more accurate diagnoses while simultaneously exploring minimally invasive interventions. This dual functionality is set to transform traditional surgical pathways, potentially reducing the need for more invasive resections and streamlining patient care. As RAB technology evolves, it may be a critical adjunct to video-assisted and robotic-assisted thoracic surgery, offering preoperative localization of small nodules and even intraoperative guidance. With ongoing research into combining ION with molecular diagnostic tools and immunotherapy delivery, robotic-assisted bronchoscopy is emerging as a key player in diagnosis and treatment. By integrating precision-guided biopsies with potential therapeutic interventions, such as localized drug delivery and targeted ablation, this technology is helping to close the gap between early diagnosis and personalized lung cancer management (14).

## Addressing barriers to adoption

Despite its advantages, the widespread adoption of the ION faces challenges, including high costs and the need for operator training. Studies have highlighted that the acquisition of the platform and its associated instruments can be a significant financial burden for institutions. Furthermore, the implementation of training programs for clinicians to master the advanced features of ION is critical, as improper usage can negate its benefits. Previous research has also pointed out variability in outcomes based on operator experience, emphasizing the need for standardized training protocols to ensure consistency and reliability in clinical practice (1, 2). The cost of using this technology is justified when applied correctly—specifically for small nodules, locations inaccessible with standard methods, and both pure ground-glass opacities (GGO) and part-solid nodules, which can be particularly challenging to biopsy using conventional techniques. However, the platform’s demonstrable impact on diagnostic outcomes justifies investment in its integration into clinical practice. As institutions like the Memorial Sloan Kettering Cancer Center in the US and Shanghai Chest Hospital in China adopt the technology, its role in standardizing care for lung cancer patients becomes increasingly apparent (5, 8).

For these reasons, the IEO, European Institute of Oncology IRCCS in Milan, became the first center in Italy, and among the leaders in Europe, to adopt ION to perform biopsies more accurately and quickly, significantly reducing the time to diagnosis and enabling timely, minimally invasive treatments. In addition to its diagnostic capabilities, ION will mark lung nodules with specific markers, such as technetium or methylene blue, making them easily identifiable for robotic minimally invasive surgery. In the future, the ION robotic arm will be equipped with a probe for thermoablation, allowing small nodules to be removed without surgery. This technique is already being explored in the United States and the United Kingdom, and IEO, European Institute of Oncology IRCCS is preparing to launch its clinical study.

## Conclusion

The ION platform represents a paradigm shift in the diagnosis and management of peripheral pulmonary lesions. Its unmatched precision, safety, and versatility make it a cornerstone in modern interventional pulmonology. As clinical experience and technological refinements accumulate, the ION is poised to lead the charge in transforming pulmonary diagnostics and therapeutics.
